# High prevalence of *Schistosoma japonicum* by perfusion in naturally exposed water buffalo in a region of the Philippines endemic for human schistosomiasis

**DOI:** 10.1371/journal.pntd.0009796

**Published:** 2021-09-16

**Authors:** Mario Jiz, Claro Mingala, Zhi-Qiang Fu, Melika Adriatico, Ke Lu, Blanca Jarilla, Marianne Sagliba, Ammabelle Moreno, Sangshin Park, Jiao-Jiao Lin, Remigio Olveda, Jonathan D. Kurtis, Hannah W. Wu

**Affiliations:** 1 Department of Immunology, Research Institute of Tropical Medicine, Manila, Philippines; 2 Philippine Carabao Center, Science City of Munoz, Nueva Ecija, Philippines; 3 Shanghai Veterinary Research Institute, Chinese Academy of Agricultural Sciences, Shanghai, China; 4 Graduate School of Urban Public Health & Department of Urban Big Data Convergence, University of Seoul, Seoul, Republic of Korea; 5 Center for International Health Research, Rhode Island Hospital, Brown University Medical School, Providence, Rhode Island, United States of America; Ghent University, BELGIUM

## Abstract

In the past decade, ecological surveys emphasized rats and dogs as the most significant animal reservoirs for *Schistosoma japonicum* (*S*.*j*) in the Philippines. However, recent studies demonstrated 51–91% prevalence of schistosomiasis among water buffalo using qPCR in the Sj endemic regions in the Philippines. In order to resolve the inconsistency of reported surveys regarding Sj endemicity among carabao, a domestic water buffalo that is the most important draught animal, we introduced 42 schistosome negative water buffalo to Macanip, Jaro municipality, Leyte, the Philippines, a subsistence rice-farming village that has been the focus of schistosomiasis japonica studies of our group for the past 20 years. We conducted perfusion to the remaining 34 buffalo that survived 10 months of nature exposure and Typhoon Haiyan. Thirty-three water buffalo were found to be positive with at least 1 pair of worms from the mesenteric vein. The infection rate is 97%, with the worm burden of 94 (95% confidence interval, 49–138 worms) worms. To our knowledge, this is the first report about *S*. *japonicum* worm burden in naturally infected water buffalo in the Philippines. The fact that with less than one-year of exposure, in this human schistosomiasis endemic area, only 1 out of 34 water buffalo was uninfected is striking. Urgent attention is needed for a cost-effective technique for monitoring *Sj* infection in animals and humans. Meanwhile, intervention implementation, including water buffalo treatment and vaccination, should be taken into consideration.

## Author summary

Schistosomiasis japonica is a zoonosis, with water buffalo (carabao) constituting a major non-human reservoir. In the Philippines, previous reports based on conventional coprological examination of carabao feces have concluded that the prevalence of *S*. *japonicum* infection is low. We now report schistosome worm burden data by perfusion from water buffalo that were previously uninfected and subsequently exposed to *Sj* by moving to highly endemic villages. Perfusion of 34 water buffalo in Macanip, Leyte, Philippines revealed that 33 out of the 34 animals (97%) had at least one pair of *S*. *japonicum* worms in the mesenteric veins after 10 months of exposure. To date, no direct worm recovery report from naturally exposed water buffalo in the Philippines has been published. Our data suggested that urgent attention is needed to qualify the integrated measurements for *Sj* infection control in the Philippines, considering water buffalo as an important drought animal and its role in human schistosomiasis transmission.

## Introduction

Schistosomiasis is a major public health concern in the developing world caused by parasitic helminths of the genus *Schistosoma*. The three major *Schistosoma* species (*S*. *japonicum*, *S*. *mansoni*, *S*. *hematobium*) infect 252 million individuals globally [1]. The Philippines is widely affected by *S*. *japonicum*, and the infection is endemic among people in 28 of its 81 provinces [2]. Two million Filipinos are directly at risk for schistosomiasis, with 12 million residing in endemic areas. Infection is mediated by contacting with slow-moving freshwater contaminated with the schistosome larvae. Schistosomiasis leads to a complicated set of morbidities ranging from anaemia, growth stunting, cognitive impairment, to liver and spleen enlargement, fibrosis, and even death when left untreated. The Philippine Department of Health spearheads the Schistosomiasis Control and Elimination Program (SCEP), which aims to eliminate schistosomiasis as a public health threat (defined as <1% prevalence in all endemic areas) by 2025. To this end, the SCEP is aggressively promoting mass drug administration (MDA) with Praziquantel, among other interventions, to control the infection.

Complicating control initiatives for schistosomiasis japonica is the zoonotic nature of the parasite. This infection persists in its intermediate host, the snail, which contributes to rapid reinfection despite the presence of MDA programs. Indeed, the Philippines has historically demonstrated that mass treatment is insufficient to sustainably control schistosomiasis: at the termination of support for aggressive case finding and treatment, prevalence rates returned to pre-treatment levels.

The carabao, a domestic water buffalo that is the essential draught animal in the Philippines, is thought to be a reservoir host for *S*. *japonicum (Sj)*. In 1950, Hunter et al. reported positive cultures of faecal samples from one out of 6 carabao in Mindoro[3]. This was the first time that *Sj* infection was reported in water buffalo. Seminal work on animal schistosomiasis in the Philippines in 1958 showed that dogs, pigs, rats, cattle and water buffalo were all infected with the parasite[4–6]. In 1981, Dumag et al published an intensive study on animal reservoir hosts of schistosomiasis in the Philippines, studying Dagami and adjacent towns of Leyte area. Faecal samples from a total of 680 dogs, 1168 pigs, 1825 carabaos, 15 cattle, 44 goats and 147 field rats were examined using the Merthiolate Iodine-Formaldehyde Concentration (MIFC) technique. The prevalence was 8.09% in dogs, 4.28% in pigs, 0.38% in carabaos, and 73.72% in rats, but 0% in cattle and goats [7].

In the past decade, ecological surveys emphasized rats and dogs as the most significant animal reservoirs for both infection burden and potential for transmission [8,9]. In contrast, reports from China have elegantly demonstrated the prominent role of water buffalo accounting for 75–93% of *Sj* transmission [10,11]. In a study in China that focused on 4 villages, elimination of water buffalo drastically reduced infection levels in humans in the village where water buffalo were replaced by mechanized farm equipment [12]. This approach is being implemented throughout endemic regions in China [13]. Our group has previously demonstrated that water buffalo from an endemic village in Leyte, Philippines, were highly infected with schistosomiasis (51.5% prevalence), as shown by a validated real-time polymerase chain reaction assay (quantitative PCR or qPCR). We argued that the low prevalence previously reported was attributable to coprological tests’ poor performance in the fiber-rich water buffalo stool [14]. In support of this hypothesis, subsequent animal surveys from the adjacent Samar province showed 81–91% prevalence of schistosomiasis among water buffalo using qPCR, which emphases the poor performance of traditional techniques [15,16]. To resolve the inconsistency of reported surveys, we conducted an incidence study of schistosomiasis in water buffalo by exposing uninfected animals to natural infection over several months. We used two methods to quantify schistosome eggs in stool samples collected longitudinally, in compare with a perfusion method to quantify worm burdens at the end. A secondary aim was to identify the most reliable and cost-effective method for monitoring *S*. *japonicum* infection in water buffalo.

## Methods

### Ethics statement

This study was approved by the Institutional Animal Care and Use Committee (IACUC) and the Institutional Review Board (IRB) of the Research Institute for Tropical Medicine (Protocol 2012–021).

### Study animals

Forty-two (42) healthy 2–3 years old native riverine water buffalo (*Bubabus bubalis*) of mixed sex were purchased from a farm in Masbate, an island in central Philippines. The animals were transported to Gandara, Northern Samar, and housed in a roofed, enclosed Philippine Department of Agriculture facility. Study animals were provided water ad libitum and fed exclusively with fresh grass collected and cut daily, supplemented with dried rice stalk, mineral block, and vitamins. The water buffalo were not allowed to graze nor leave the enclosed premises. A veterinarian was on-site during the study period to monitor animal health. All 42 study animals were treated with 25 mg/kg Praziquantel [17] upon arrival and after four months in Gandara, just prior to transport in October 2013, to Macanip village in Jaro, Leyte, an area endemic for schistosomiasis. Animals were distributed to 21 farmer-recipients, who freely used the animals for rice farming, coconut harvesting, transportation, and other means. Stool samples were collected from study animals before releasing to the farmers and monthly after arrival in Macanip (April to July 2014 for Month 6 to 9). In August 2014, 10 months after natural exposure, the animals were transported to the Ormoc City slaughterhouse, sacrificed, and perfused to quantify adult worm burdens.

### Stool examination

#### Sample processing for Formalin-ethyl acetate sedimentation (FEASD)[18]

Briefly, fifty (50) grams of fresh intrarectally collected stool was sieved in tap water through a rough and then a fine mesh until the filtrate became clear. The fine mesh retentate was washed thrice in 50 ml saline by sedimentation, a 5 ml aliquot obtained for qPCR, and the remainder was washed twice in 10% formalin. Two 5 mL aliquots were treated with ethyl acetate, centrifuged, and the debris layers discarded. The pellets were then digested overnight in 10% KOH at 37°C, centrifuged, washed in water, and resuspended in 4 mL of 10% formalin. 100 uL aliquots were examined under the microscope, and *S*. *japonicum* eggs quantified by trained microscopists. A water buffalo is considered cumulated positive by FEASD if EPG>0 was identified by any of the stool exams after exposure. The cumulated EPG is calculated as the mean of all EPGs detected during 6 to 9 months’ exposure, missing data points are excluded.

#### Sample processing for qPCR

The 5 mL stool qPCR aliquot was spun down, resuspended in 70% ethanol, and transported to Manila for DNA extraction and qPCR. Stool suspension of 1mL was spun down, and the stool pellet was adjusted to 200 mg. DNA was extracted using the Qiagen Stool DNA extraction kit with modifications. Upon addition of 1.4 mL Buffer ASL, samples were sonicated in an ice bath for 4x30 seconds using a probe sonicator with 15-sec rest in between runs. Samples were then incubated in a boiling water bath for 20 minutes, cooled, and the InhibitEX tablet added. DNA was extracted following manufacturer’s instructions using overnight alcohol precipitation. Real time PCR was performed as described by Wu et al [14]. Briefly, we amplified the *S*. *japonicum* specific 82-base-pair sequence in the mitochondrial nicotinamide adenine dinucleotide dehydrogenase I (NADH-1) gene using SYBR Green for quantification. The PCR program was 95.0°C for 15 minutes; 40 cycles at 95.0°C for 15 seconds and 60.0°C for 1 minute; and a melting point analysis of 60.0–95.0°C over 15 minutes by using Applied Biosystems 7500 Real-Time PCR System (Thermo Fisher Scientific, USA). DNA extracted from spiked stool samples with 1, 2, 5, 10, 20, 40, 80 EPG were amplified in triplicate with each assay to construct a standard curve for quantifying unknown samples. The PCRs for all unknown samples were carried out in duplicates. The estimated EPG by qPCR was calculated based on the standard curve for each plate and recorded as the average value of the duplicate assays. A water buffalo is considered cumulated positive by qPCR if EPG>0 was identified by any of the stool exams after exposure. The cumulated EPG is calculated as the mean of all EPGs detected during 6 to 9 months’ exposure, missing data points are excluded.

### Perfusion

Water buffalo were stunned in the occipital hole with a sharp knife. After incision at the left carotid artery, the animal was bled fully. Subsequent steps ensured that the vasculature remained intact. The skin was removed, and the chest and abdominal cavities were exposed by severing the sternum and removing the ribs. The metal perfusion tube was inserted and secured in the upper third of the thoracic aorta, and the pulmonary artery severed and blocked with a hemostat. After cutting the peritoneum, the renal arteries and veins, side iliac arteries and veins, and bile duct were all blocked using hemostats. The hepatic portal vein was clamped at the distal and proximal end, and a metal sieve was placed below the distal. Tap water flowed into the perfusion tube connected with the thoracic aorta, and worms collected through the sieve. The small intestines and colon were continuously agitated during perfusion to dislodge worms from the mesenteric veins. Then, worms from the portal vein were collected by inserting the perfusion tube in the pulmonary artery and opening the proximal end of the hepatic portal vein. The liver of each carabao was separated from the body and examined for the remaining schistosome and *Fascioloa* worms. The *Sj* adult worms were enumerated by three independent readers. The presence of *Fasciola* worms was recorded.

### Data management and statistical analysis

Data were encoded in password protected Filemaker Pro12 databases. Descriptive statistics and Spearman correlation analysis between worm burden and EPG by FEASD and qPCR were performed using either GraphPad Prism v9 (GraphPad Software Inc, La Jolla, CA) or SAS (v.9.4, SAS Institute, Cary, NC).

## Results

Forty-two healthy water buffalo uninfected with *Sj* as confirmed by FEASD and qPCR (S1A Table), were distributed to farmer-recipients for draft animal use in Macanip village, Jaro, Leyte in October 2013. In November 2013, the study was disrupted because the island of Leyte was devastated by super typhoon Haiyan, which halted all field operations. We were not able to properly examine the stool samples collected 1–5 months after animals were released to recpient farmers. Activities resumed in April 2014 after reconstruction and rehabilitation of damaged research facilities in Palo, Leyte. Eight animals were lost in the storm and in the subsequent months.

**Fig 1:**
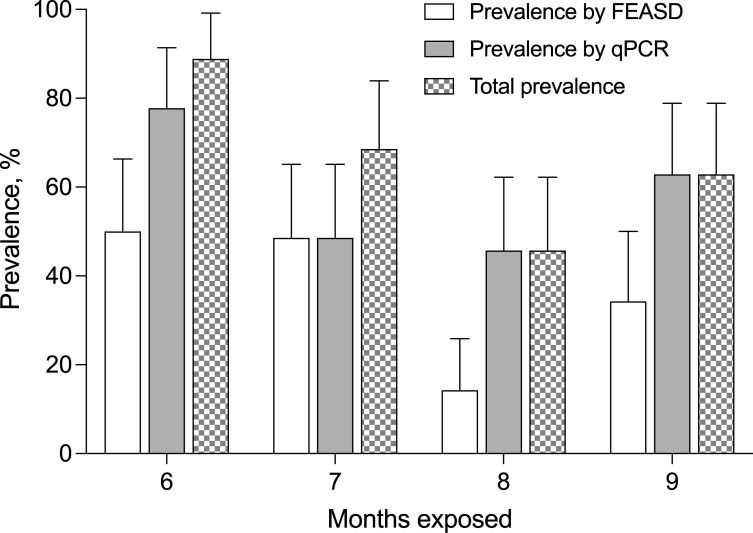
Prevalence of S. japonicum in water buffalo using coprological FEASD and real-time PCR techniques at six to nine months post-natural exposure. Total prevalence incorporates both FEASD & qPCR results. Error bars represent 95% confidence intervals.

**Fig 2:**
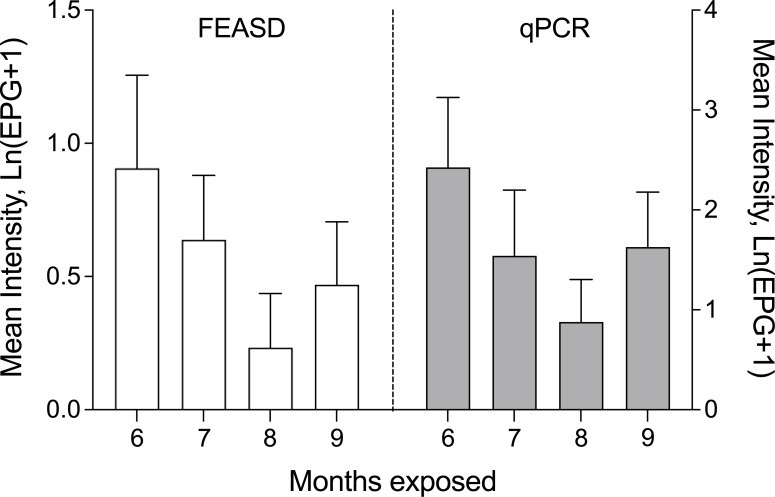
Natural log transformed intensity of *S*. *japonicum* infection among water buffalo by FEASD& qPCR at 6 to 9 months post-exposure. Error bars represent 95% confidence.

Six months of natural exposure revealed the prevalence of schistosomiasis in the remaining animals to be 77.8% by PCR and 50.0% by FEASD (Fig 1). In combined qPCR & FEASD analysis, the total prevalence of schistosomiasis was 88.9%. Fecal egg burden measures (namely EPG, which is eggs per gram feces) were consistently higher in qPCR compared to FEASD at all timepoints (Natural log transformed mean EPG of 10.3 vs 1.5 at 6 months, Fig 2 and S1B Table). Subsequent monthly stool analyses showed fluctuations in the prevalence and intensities of infection. At 7 months of natural exposure, there was a 48.6% prevalence for both qPCR & FEASD and a combined 68.6% prevalence. Egg burdens similarly fluctuated to 3.7 by qPCR and 0.9 by FEASD. At 8 and 9 months of exposure, qPCR detected all FEASD-positive animals, with a prevalence of 45.7% and 64.7%, respectively. Egg burdens were lowest at 8 months of exposure (1.4 epg by qPCR, 0.26 epg by FEASD), but rebounded at 9 months (4.1 epg by qPCR, 0.60 by FEASD).

**Fig 3:**
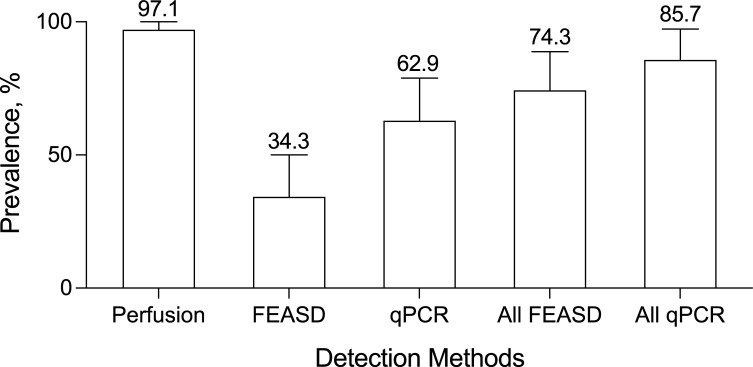
High prevalence of schistosomiasis japonica in water buffalo as assessed by animal perfusion in comparison to coprological techniques. S. japonicum prevalence by perfusion (n = 34) of adult worms from the mesenteric veins at 10 months post-exposure, in comparison to coprological FEASD (n = 35), and qPCR (n = 35), techniques. Error bars represent 95% confidence intervals.

Perfusion of water buffalo after 10 months revealed that 33 out of the 34 animals had adult schistosome worms, corresponding to a 97.1% incidence of schistosomiasis among water buffalo at 10 months of natural exposure in a highly endemic village (Fig 3 and S1A Table). Mean worm burden was 94 worms per animal (95% confidence interval, 49–138 worms). Using perfusion results as the reference, sensitivity of stool examination after 9 months exposure is 33.3% by FEASD, and 63.6% by qPCR. When counting all positive stool results between 6–9 months exposure, the sensitivity increased to 75.8% by FEASD, and 87.9% by qPCR. The sensitivity is the highest at 93.9% when all the stool FEASD and qPCR results were combined. The water buffalo having only 1 pair of worms by perfusion tested negative at all timepoints by both methods (S1A Table). An additional finding from the perfusion analyses went beyond our original study design. We found that the animals were consistently coinfected with large numbers of *Fasciola* worms. The coinfection rate was 97.1% (S1C Table).

**Fig 4:**
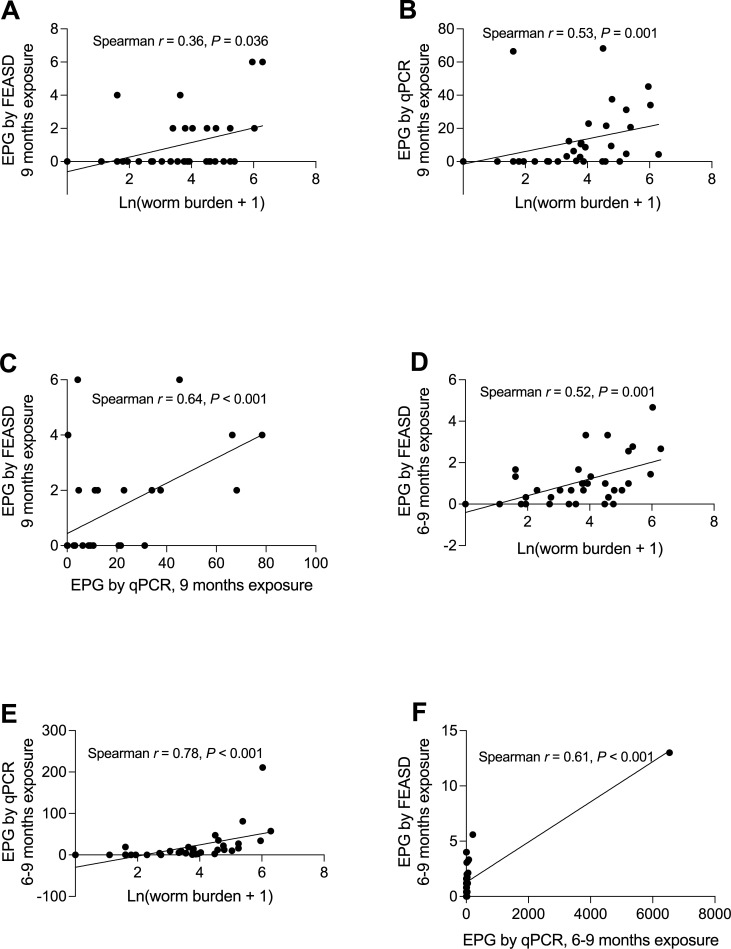
Correlation between coprological techniques and worm burden by perfusion. A, B and C showed 9 months’ stool EPG by FEASD and qPCR. D, E and F showed cumulated EPG over 6–9 months and the respective correlation.

To identify the most reliable and cost-effective method for monitoring *S*. *japonicum* infection in water buffalo. We looked at the correlation of EPG by FEASD and qPCR, as well as their relationship with the number of worms by perfusion (Fig 4). Worm burdens significantly correlated with EPG by qPCR (Spearman’s Rho = 0.53, p-value <0.001). Correlation to worm burden improved when all longitudinal stool results for both FEASD (Fig 4A vs. 4D) and qPCR (Fig 4B vs. 4E) were combined. EPGs by the 2 coprological detection methods are significantly correlated (Fig 4C and 4F).

## Discussion

The role of water buffalo in the transmission of schistosomiasis in the Philippines has been debated, given the conflicting results of previous reports relying on coprological tests, which showed very low prevalence (0.38%) [7]. Newer studies using molecular tests showed prevalence from 51 to 91% prevalence [2,14–16,19]. To our knowledge, this is the first report quantifying actual worm burdens measured by perfusion among water buffalo naturally exposed to schistosomiasis in the Philippines. Previously reported worm burdens in dogs and pigs showed a range from 2–156 worms per animal [7], while we found a burden of 2–537 worms in water buffalo. This work is pivotal in confirming the strikingly high burden of disease among water buffalo in the Philippines. In China, water buffalo are known to be the major source of schistosomiasis transmission. Thus, our study demonstrates the importance of schistosomiasis control in water buffalo to protect human health in the Philippines.

Several reasons may account for the major role of water buffalo in schistosomiasis. First, these animals release a large volume of faecal matter into the environment, averaging 20 kg daily. Second, these animals are dependent on wallowing in water or mud to cool down from the tropical heat, thus their affinity to water sources poses a great risk of infecting the amphibious snail hosts that cohabit the area. Third, because of their importance as draught animals, frequent interaction between humans and water buffalo occurs, potentially increasing the risk of human water contact to schistosome-contaminated water Finally, because water buffalo infected with schistosomiasis show few if any signs of morbidity, infected animals continue to be used by farmers, thereby sustaining the infection in the environment.

The almost universal (97%) schistosomiasis infection among water buffalo within a short period (10 months) in this study unequivocally demonstrates the high burden of schistosomiasis in this animal reservoir. The findings support our previous work demonstrating the high schistosomiasis endemicity of the Macanip village [14]. In an immuno-epidemiologic survey conducted in 2002 among 7–30 year-old residents of the same village, the prevalence was found to be 60% by duplicate Kato-Katz examination of 3 stool samples [20]. Reinfection was likewise rapid, and the adjusted mean time to reinfection for an average participant in the same cohort was 8.6 months using a proportional hazards modeling [21]. In 2012, 38% of schoolchildren aged 7–16 studying in the village elementary school were infected with schistosomiasis (M. Jiz, ASTMH abstract book 2013# 425 https://mesamalaria.org/sites/default/files/2018-12/ASTMH%20Abstract%20book%202013.pdf). Overall, these data suggest a dynamic persistence of infection in the environment, likely contributable to the animal reservoirs, since the compliance is high. The high prevalence of infection and reinfection in humans despite treatment suggests that control of schistosomiasis in water buffalo should be integrated into public health policy.

Results of the worm burden count by perfusion, and either FEASD or qPCR techniques which indicated much lower egg counts, highlight the limitations of using stool examinations to approximate the infection status of water buffalo. We found that the FEASD sedimentation technique, and surprisingly even qPCR, grossly underestimated the true prevalence based on actual worm burdens. We report sensitivities of 33.3% for FEASD and 63.6% for qPCR on stool samples collected just prior to perfusion. However, the strong (p<0.001) correlation between qPCR egg burdens and perfusion worm counts is heartening, suggesting the potential of qPCR to quantify intensity of infection. Our data also showed that when the results of coprological testing among longitudinal collected samples were combined, the detection sensitivity improved dramatically by either FEASD or qPCR. The inconsistent and non-random distribution of schistosome eggs in the stool in water buffalo, as in humans, may play a role in this under-measurement by non-perfusion methods. In addition, the low *S*.*japonicum* infection intensity among the study water buffalo may contribute to the lower sensitivity of stool examinations [22,23]. In our study, only 0.1% of average daily discharged (about 50 kg) stool were collected from each animal at a given day account for the fluctuation of prevalence and EPGs at different timepoints. It is necessary to collect multiple stool samples over timepoints and on different days from water buffalo, as is done with human surveys [24]. However, this approach will be expensive, given the high price of stool DNA extraction and qPCR assays. Development of more sensitive and cost-effective diagnostics for animal schistosomiasis is therefore clearly needed.

We recognize that super typhoon Haiyan may have altered vector distribution during the study period and thereby contributed to high infection rates. However, reports from the adjacent Samar provinces prior to November 2013 have also shown an up to 90% prevalence in water buffalo by stool qPCR [15], suggesting that high rates of water buffalo infection may be common in the schistosomiasis endemic regions in the Philippines. The study’s finding of a high prevalence of schistosomiasis in water buffalo is potentially limited by the fact that the study animals were purchased from a high endemic region. Hence, they may have had residual infection despite our pre-treatment [17] and stool tests to verify the non-infected status before the study began.

Control of animal schistosomiasis, particularly in water buffalo, is essential to sustainably control human infection in the Philippines. Further studies on the direct contribution of water buffalo in schistosomiasis transmission to humans are needed. Interventions to eliminate water buffalo in endemic areas in the Philippines may well help to protect human health. Alternative control strategies that target animal reservoirs, including vaccination and treatment, offer great promise [13].

In the Philippines, the presence of bovine fasicioliasis has been documented [25], because water buffalo are disabled by the liver flukes and causes economic loss [26]. In contrast, *S*. *japonicum* infection in water buffalo has not been monitored and treated. This policy ignores the economic impact of schistosomiasis in humans, whose productivity is reduced by the disease transmission from water buffalo. Nevertheless, it has been recently argued that animal schistosomiasis likely causes a considerable drain both directly and indirectly on local economy [27]. We have demonstrated a high level of coinfection of *Fasciola* and *S*. japonicum with 97% of carabaos harbouring both species. These data support a co-treatment approach to protect the water buffalo from fascioliasis and decrease their contribution to human schistosomiasis transmission.

In conclusion, through controlled nature exposure and animal perfusion, we found a very high prevalence of *S*. *japonicum* infection in water buffalo in an endemic region of the Philippines. We demonstrated that stool examinations by 2 methods were not very sensitive, compared to perfusion analysis of worm burden, possibly due to the low intensity of infection among the water buffalo. Cost-effective technique for monitoring *S*.*japonicum* infection in both animals and humans is urgently needed. Although MDA to humans in a community is effective in curing the disease, reinfection is extremely common because humans are exposed through the presence of reservoir hosts such as water buffalo. Hence, in the Philippines, control measurement of schistosomiasis in water buffalo is essential to protect human health and support a strong economy.

## Supporting information

S1 Table A. Individual data for diagnostic methods at each time point. Perfusion happened 2.5wks after 9 months exposure. B. Summary of *S*. *japonicum* intensity by FEASD and qPCR detection method at each stool collection timepoint. C. Summary of the Fasciola eggs by FEASD. The symbol “/” is to show the missing data.
(DOCX)
