# Supplementary material for: High prevalence of Schistosoma japonicum by perfusion in naturally exposed water buffalo in a region of the Philippines endemic for human schistosomiasis
Source: PLoS Negl Trop Dis. 2021 Sep 16;15(9):e0009796. doi: 10.1371/journal.pntd.0009796 (PMC8478178; doi:10.1371/journal.pntd.0009796)
Supplement: S1 Table — A. Individual data for diagnostic methods at each time point. Perfusion happened 2.5wks after 9 months exposure. B. Summary of S. japonicum intensity by FEASD and qPCR detection method at each stool collection timepoint. C. Summary of the Fasciola eggs by FEASD. The symbol “/” is to show the missing data. (DOCX) [file pntd.0009796.s001.docx]

**Table A.** Individual data for diagnostic methods at each time point. Perfusion happened 2.5wks after 9 months exposure. The symbol “/” is to show the missing data.

| **ID** | **Worm counts by Perfusion** | | |  | **EPG by FEASD months after exposure** | | | | |  | **EPG by qPCR months after exposure** | | | | |
| --- | --- | --- | --- | --- | --- | --- | --- | --- | --- | --- | --- | --- | --- | --- | --- |
|  | **Paired (n=34)** | **Male (n=34)** | **Worm burden (n=34)** |  | **0 (n=42)** | **6 (n=36)** | **7 (n=35)** | **8 (n=35)** | **9 (n=35)** |  | **0 (n=42)** | **6 (n=36)** | **7 (n=35)** | **8 (n=35)** | **9 (n=35)** |
|  |  |  |  |  |  |  |  |  |  |  |  |  |  |  |  |
| A01 | 75 | 3 | 153 |  | 0 | 4 | 0 | 0 | 0 |  | 0 | 25 | 7 | 8 | 0 |
| A02 | 264 | 9 | 537 |  | 0 | 8 | 2 | 0 | 6 |  | 0 | 76 | 150 | 0 | 4 |
| A03 | 2 | 1 | 5 |  | 0 | 0 | 0 | 0 | 0 |  | 0 | 0 | 0 | 0 | 0 |
| A04 | 93 | 3 | 189 |  | 0 | 6 | 0 | 0 | 0 |  | 0 | 24 | 51 | 3 | 31 |
| A05 | / | / | / |  | 0 | / | / | / | / |  | 0 | / | / | / | / |
| A06 | 15 | 4 | 34 |  | 0 | 0 | 0 | 0 | 0 |  | 0 | 11 | 0 | 0 | 6 |
| A07 | 2 | 0 | 4 |  | 0 | 0 | 4 | 0 | 4 |  | 0 | 2 | 0 | 8 | 66 |
| A08 | 18 | 1 | 37 |  | 0 | 2 | 0 | 0 | 4 |  | 0 | 13 | 48 | 14 | 0 |
| A09 | 7 | 1 | 15 |  | 0 | 0 | 2 | 0 | 0 |  | 0 | 0 | 0 | 0 | 0 |
| A10 | 13 | 1 | 27 |  | 0 | 0 | 0 | 0 | 0 |  | 0 | 9 | 0 | 11 | 3 |
| A11 | / | / | / |  | 0 | / | / | / | / |  | 0 | / | / | / | / |
| A12 | 189 | 8 | 385 |  | 0 | 0 | 0 | 3 | 6 |  | 0 | 37 | 0 | 55 | 45 |
| A13 | 4 | 1 | 9 |  | 0 | 2 | 2 | 0 | 0 |  | 0 | 0 | 0 | 0 | 0 |
| A14 | / | / | / |  | 0 | 26 | / | / | / |  | 0 | 6539 | / | / | / |
| A15 | 3 | 0 | 6 |  | 0 | 0 | 0 | 0 | 0 |  | 0 | 0 | 0 | 0 | 0 |
| A16 | 102 | 13 | 217 |  | 0 | 12 | 0 | 3 | 0 |  | 0 | 249 | 49 | 6 | 21 |
| A17 | 57 | 2 | 116 |  | 0 | 0 | 0 | 0 | 0 |  | 0 | 37 | 43 | 0 | 9 |
| A18 | 20 | 2 | 42 |  | 0 | 4 | 2 | 0 | 0 |  | 0 | 0 | 0 | 0 | 3 |
| A19 | 22 | 3 | 47 |  | 0 | 16 | 4 | 0 | 0 |  | 0 | 7 | 0 | 0 | 0 |
| A20 | 44 | 0 | 89 |  | 0 | 0 | 4 | 0 | 2 |  | 0 | 20 | 101 | 0 | 68 |
| A21 | 3 | 0 | 6 |  | 0 | 2 | 0 | 0 | 0 |  | 0 | 0 | 0 | 1 | 0 |
| A22 | 46 | 3 | 96 |  | 0 | 10 | 4 | 0 | 0 |  | 0 | 40 | 3 | 6 | 0 |
| A23 | / | / | / |  | 0 | / | / | / | / |  | 0 | / | / | / | / |
| A24 | / | / | / |  | 0 | / | / | / | / |  | 0 | / | / | / | / |
| A25 | 0 | 0 | 0 |  | 0 | 0 | 0 | 0 | 0 |  | 0 | 0 | 0 | 0 | 0 |
| A26 | / | / | / |  | 0 | 2 | 2 | 3 | 4 |  | 0 | 63 | 75 | 24 | 78 |
| A27 | 27 | 1 | 55 |  | 0 | 4 | 2 | 0 | 2 |  | 0 | 0 | 0 | 1 | 23 |
| A28 | 59 | 1 | 119 |  | 0 | 0 | 2 | 0 | 2 |  | 0 | 12 | 0 | 0 | 38 |
| A29 | 48 | 2 | 99 |  | 0 | 0 | 0 | 0 | 0 |  | 0 | 95 | 24 | 0 | 22 |
| A30 | 205 | 2 | 412 |  | 0 | 10 | 8 | 8 | 2 |  | 0 | 549 | 232 | 28 | 34 |
| A31 | 1 | 0 | 2 |  | 0 | 0 | 0 | 0 | 0 |  | 0 | 0 | 0 | 0 | 0 |
| A32 | / | / | / |  | 0 | / | / | / | / |  | 0 | / | / | / | / |
| A33 | 21 | 1 | 43 |  | 0 | 2 | 4 | 0 | 0 |  | 0 | 0 | 18 | 0 | 11 |
| A34 | 41 | 5 | 87 |  | 0 | 0 | 0 | 0 | 0 |  | 0 | 11 | 0 | 0 | 0 |
| A35 | 93 | 3 | 189 |  | 0 | 6 | 2 | 5 | 2 |  | 0 | 17 | 38 | 5 | 5 |
| A36 | 21 | 2 | 44 |  | 0 | 0 | 2 | 0 | 2 |  | 0 | 17 | 24 | 7 | 11 |
| A37 | 14 | 1 | 29 |  | 0 | 0 | 2 | 0 | 2 |  | 0 | 26 | 0 | 0 | 12 |
| A38 | 2 | 0 | 4 |  | 0 | 0 | 0 | 0 | 0 |  | 0 | 2 | 0 | 0 | 0 |
| A39 | 25 | 0 | 50 |  | 0 | 4 | 0 | 0 | 0 |  | 0 | 2 | 0 | 0 | 9 |
| A40 | / | / | / |  | 0 | / | / | / | / |  | 0 | / | / | / | / |
| A41 | 7 | 0 | 14 |  | 0 | 0 | 0 | 0 | 0 |  | 0 | 13 | 3 | 0 | 0 |
| A42 | 10 | 0 | 20 |  | 0 | 2 | 2 | 0 | 0 |  | 0 | 36 | 2 | 0 | 0 |

**Table B.** Summary of *S. japonicum* intensity by FEASD and qPCR detection method at each stool collection timepoint.

|  | **EPG by FEASD months after exposure** | | | |  | **EPG by qPCR months after exposure** | | | |
| --- | --- | --- | --- | --- | --- | --- | --- | --- | --- |
|  | **6** | **7** | **8** | **9** |  | **6** | **7** | **8** | **9** |
| **Transformed mean EPG** | 1.474 | 0.893 | 0.262 | 0.598 |  | 10.322 | 3.673 | 1.406 | 4.093 |
| **Lower 95% CI of EPG** | 0.743 | 0.486 | 0.030 | 0.262 |  | 4.623 | 1.422 | 0.573 | 1.938 |
| **Upper 95% CI of EPG** | 2.511 | 1.409 | 0.547 | 1.024 |  | 21.798 | 8.014 | 2.680 | 7.827 |

**Table C.** Summary of the Fasciola eggs by FEASD . The symbol “/” is to show the missing data.

| **ID** | **EPG by FEASD months after exposure** | | | | |  |
| --- | --- | --- | --- | --- | --- | --- |
|  | **0 (n=42)** | **6 (n=36)** | **7 (n=35)** | **8 (n=35)** | **9 (n=35)** |  |
|  |  |  |  |  |  |  |
| A01 | 2 | 48 | 20 | 32 | 32 |  |
| A02 | 2 | 118 | 36 | 64 | 28 |  |
| A03 | 0 | 152 | 130 | 46 | 110 |  |
| A04 | 20 | 120 | 26 | 30 | 26 |  |
| A05 | 2 | / | / | / | / |  |
| A06 | 0 | 272 | 170 | 76 | 156 |  |
| A07 | 0 | 98 | 32 | 36 | 40 |  |
| A08 | 0 | 34 | 46 | 8 | 26 |  |
| A09 | 4 | 82 | 14 | 28 | 14 |  |
| A10 | 2 | 14 | 4 | 20 | 22 |  |
| A11 | 0 | / | / | / | / |  |
| A12 | 6 | 6 | 8 | 22 | 30 |  |
| A13 | 18 | 10 | 92 | 104 | 50 |  |
| A14 | 2 | 8 | / | / | / |  |
| A15 | 6 | 54 | 212 | 56 | 112 |  |
| A16 | 4 | 26 | 32 | 36 | 46 |  |
| A17 | 6 | 16 | 24 | 16 | 62 |  |
| A18 | 6 | 16 | 28 | 24 | 16 |  |
| A19 | 26 | 42 | 24 | 50 | 14 |  |
| A20 | 0 | 30 | 62 | 22 | 38 |  |
| A21 | 6 | 10 | 50 | 44 | 36 |  |
| A22 | 20 | 8 | 42 | 28 | 14 |  |
| A23 | 4 | / | / | / | / |  |
| A24 | 4 | / | / | / | / |  |
| A25 | 12 | 28 | 32 | 24 | 30 |  |
| A26 | 4 | 80 | 74 | 28 | 60 |  |
| A27 | 2 | 64 | 30 | 28 | 74 |  |
| A28 | 0 | 56 | 12 | 30 | 6 |  |
| A29 | 0 | 52 | 14 | 24 | 48 |  |
| A30 | 4 | 34 | 20 | 68 | 68 |  |
| A31 | 4 | 58 | 62 | 40 | 48 |  |
| A32 | 0 | / | / | / | / |  |
| A33 | 8 | 94 | 2 | 14 | 26 |  |
| A34 | 0 | 162 | 186 | 38 | 36 |  |
| A35 | 0 | 110 | 54 | 46 | 42 |  |
| A36 | 4 | 238 | 68 | 10 | 32 |  |
| A37 | 2 | 62 | 10 | 28 | 32 |  |
| A38 | 0 | 32 | 12 | 50 | 70 |  |
| A39 | 2 | 54 | 8 | 24 | 20 |  |
| A40 | 0 | / | / | / | / |  |
| A41 | 2 | 140 | 26 | 30 | 16 |  |
| A42 | 6 | 90 | 170 | 48 | 122 |  |
